# Post-infectious glomerulonephritis presenting as acute renal failure in a patient with Lyme disease

**DOI:** 10.12861/jrip.2014.07

**Published:** 2013-11-08

**Authors:** Davide Rolla, Novella Conti, Francesca Ansaldo, Laura Panaro, Tiziano Lusenti

**Affiliations:** ^1^Division of Nephrology, IRCCS San Martino-IST, Genoa, Italy; ^2^Centro Medico Lazzaro Spallanzani, Reggio Emilia, Italy

**Keywords:** Lyme disease, Acute renal failure, Glomerulonephritis

## Abstract

** Introduction:** We report a case of a patient with acute renal failure in Lyme disease-associated focal proliferative mesangial nephropathy. Lyme disease is a vector-borne disease caused by Borrelia burgdorferi, transmitted by the bite of an infected ixodes tick. Post-infectious glomerulonephritis (GN)secondary to Borrelia burgdorferi infection in man could be fatal, as it is in canine Lyme borreliosis.

** Case:** A 61-year old man with chronic ethanolic hepatitis was admitted to a provincial hospital, complaining of nausea, diarrhoea and loss of his sense of taste. A few days prior hospitalization, he had been bitten by a tick. He developed erythema gyratum repens in the right leg, thorax and face. Kidney function was altered despite normal urine flow: creatinine 5.04 mg/dl and BUN 126 mg/dl. Urinalysis showed light proteinuria and microscopic hematuria. IgG and IgM antibodies to Borrelia burgdorferi were detected by ELISA and Western blot confirmed the diagnosis. Renal biopsy showed mild mesangial proliferation and mesangial and paramesangial deposits on AFOG stain. A diagnosis of acute renal failure in Lyme disease-associated focal proliferative IgA nephropathy was made. Intravenous antibiotic medication was started (ceftriaxone 1 gram daily i.v.). The patient was later discharged, serum creatinine had decreased to 3.5 mg/dl with a BUN of 58 mg/dl, and a slight improvement was observed on follow-up.

** Conclusion:** Borrelia burgdorferi is a possible cause of post-infectious GN in humans as it is in dogs. Difficulties in identifying Borrelia burgdorferi may also be one of the reasons for the paucity of reports on the association of this infection with glomerulonephritis in humans. Currently, various types of histological renal lesions have been reported.

**Implication for health policy/practice/research/medical education:**

The diagnosis of Lyme disease is based primarily on clinical history and on the presence of antibodies (IgM an IgG) to Borrelia burgdorferi. This vector-borne disease is a possible cause of post-infectious glomerulonephritis (GN) in humans as it is in dogs. It is likely that the immune-complex plays a role in the pathogenesis of this disorder, but acute Lyme disease may also contribute to the development of activation of previously immune-mediated glomerular disease.

## Introduction

Lyme disease is the most common vector-borne disease in the United States, and several species of the genus ixodes are implicated in most European cases. The causative spirochete, Borrelia burgdorferi, is transmitted by the bite of an infected ixodes tick. If left untreated the infection may lead to localized arthritis, disorders of the nervous system and even cardiac arrest. The spirochete is known to induce glomerulonephritis in animals, as has been reported in dogs (1,2). Lyme nephritis secondary to Borrelia burgdorferi infection in man could be fatal, as it is in canine Lyme borreliosis (3). To date, post-infectious glomerulonephritis (GN) associated with Lyme disease in humans has been reported four times in the literature (4-7). We report a case of a patient with acute renal failure secondary to focal proliferative IgA nephropathy, who had serologically-confirmed Lyme disease and was treated accordingly (8). It is likely that the immune-complex plays a role in the pathogenesis of this disorder, but acute Lyme disease may also contribute to the development of activation of previously immune-mediated glomerular disease.

## Case Presentation

A 61-year old man with chronic ethanolic hepatitis associated with a history of alcohol abuse was admitted to a provincial hospital in May 2009, complaining of nausea, diarrhoea and loss of his sense of taste. A few days prior to being hospitalized, he had been bitten by a tick while working in the woods. He soon developed erythema gyratum repens in the right leg, thorax and face. He also suffered joint pain in his knees, ankles and elbows. No fever or headaches were reported. At admission he suffered from asthenia, weariness and dizziness. Physical examination revealed light hypertension (150/85 mmHg), no rash, but showed pre-tibial oedema. Laboratory tests revealed an increase in erythrocyte sedimentation rate (ESR) 72 mm/h and C reactive protein (CRP) 3.2 mg/dl (N<0.5). White blood cell count was 15.42 x1000/uL, platelets were 125 x1000/uL and hemoglobin levels were 9.9 g/dl. Hepatic and pancreatic lab tests were abnormal, with gamma-GT values of 361 UI (N 8-60), alkaline phosphatase levels of 195 (N 40-129) and amylase levels of 200 UI (N 8-100). Kidney function was altered despite normal urine flow; creatinine 5.04 mg/dl and blood urea nitrogen (BUN) 126 mg/dl. Severe metabolic acidosis was observed; pH 7.26, HCO3^-^ 6.7 mmol/L, BE -17.5 mmo/L associated with hyperkalemia (6.4 mEq/l). Urinalysis showed normal density and acidity (1011 and pH 5, respectively) and microscopic hematuria. Circulating immune complexes level was slightly increased (6.5 micg/ml, N 0-3). IgG and IgM antibodies to Borrelia burgdorferi were detected by ELISA at levels of 33 U/ml (N 0-5 U/ml) and 0.210 U/ml (index 0-0.199), respectively. Western blot confirmed IgG 44 U.A. (positive score >7) and IgM 13 U.A. (positive score >7) and was interpreted as indicating present or past Lyme disease. The main laboratory parameters are listed in Table 1.

**Table 1:** Laboratory tests (at presentation)

**Parameters**	**Values**
Haematocrit (%)	30
Haemoglobin (g/dl)	9.9
White blood cell count (x10^3^)	15.4
Platelets (x1000)	125
Fibrinogen (mg/dl)	405
Prothrombin time (sec)	11
Thromboplastin partial time (sec)	34
Serum creatinine (mg/dl)	5.04
Blood urea nitrogen (mg/dl)	126
Aspartate aminotransferase (IU)	42
Alanine aminotransferase (IU)	83
Erythrocyte sedimentation rate (mm/h)	72
C-reactive protein (mg/L)	32
Bacterial Infection Markers	
Borrelia burgdorferi IgG	
ELISA (U/ml)	33
Western blotting	p10016, VisE8 p39 16
Borrelia burgdorferi IgM	
ELISA Index	0.210
Western blotting	visE6, p396, p41/i1

Kidney ultrasound showed two normal sized, hyperechoic kidneys (bipolar diameter= 10 cm; cortex <1 cm). Sodium bicarbonate and 2 blood transfusions were administered. The patient was then transferred to our unit. Serological tests for HIV, hepatitis B surface Ag and hepatitis C were negative. ANA test, extractable nuclear antigens, c-ANCA were also negative. IgA were high (1061 mg/dl; N 68-378), while IgG, IgM, C3 and C4 were within normal range. Proteinuria was 566 mg/24 h, urine volume 2000 ml/day. Mild alteration of liver profile (total bilirubin 1.2 mg/dl, GOT 42 IU, GPT 59 UI, AP 535 U/l), albumin 2.8 g/dl, CHE 1827 UI, anemia (Hb 10 g/dl, Ht 30%) and metabolic acidosis (pH 7.35, HCO3- 18 mmol/L, BE – 6.8 mEq/L) were confirmed. Intravenous antibiotic medication was started (ceftriaxone 1 gram daily i.v.) and erythropoetin (EPO) treatment (2000 U/week) was associated.

**Figure 1 (A & B):**
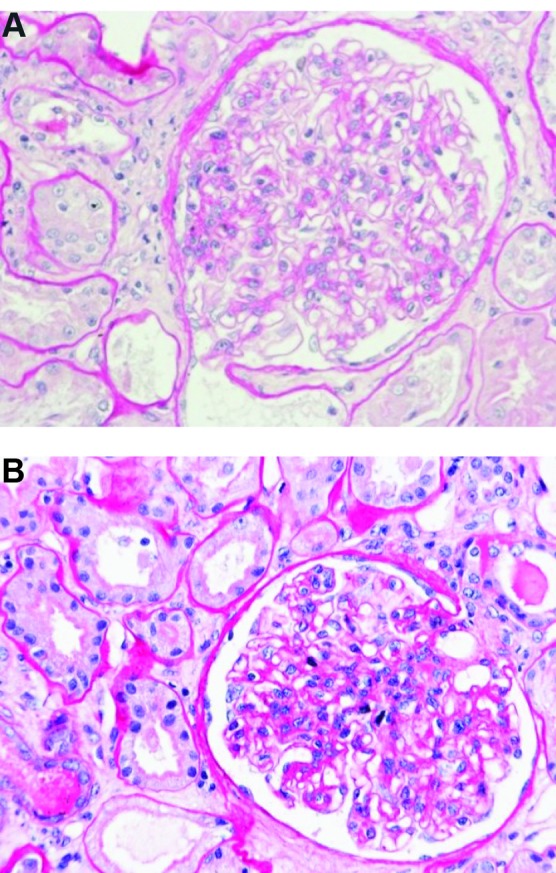
LM: mesangial expansion and mild mesangial proliferation of glomerular tuft (PAS x400)

**Figure 2:**
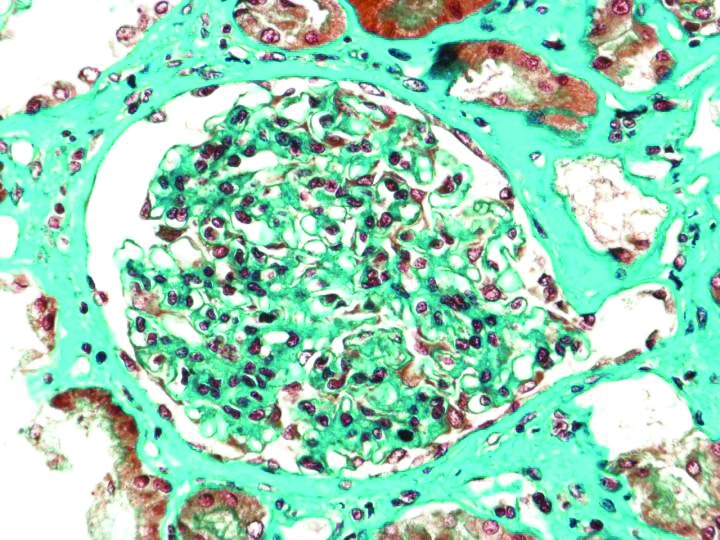
LM: mesangial expansion and global hypercellularity (Masson-Goldner trichrome x400)

**Figure 3:**
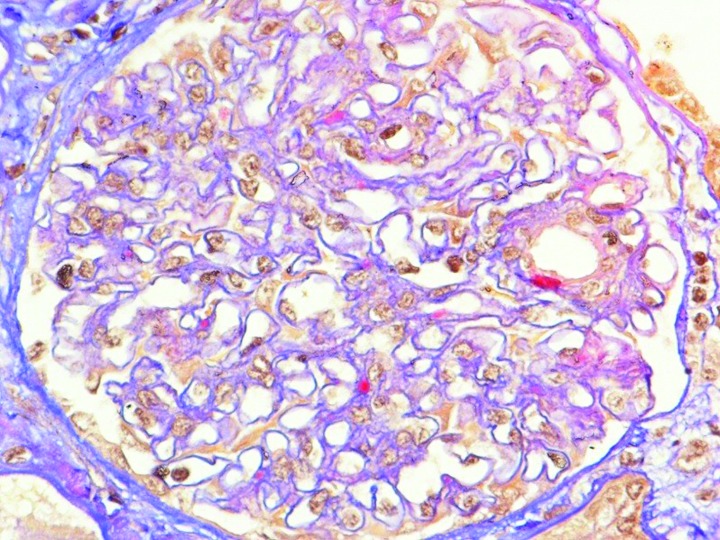
LM: mesangial and paramesangial deposits [AFOG stain x400]

**Figure 4:**
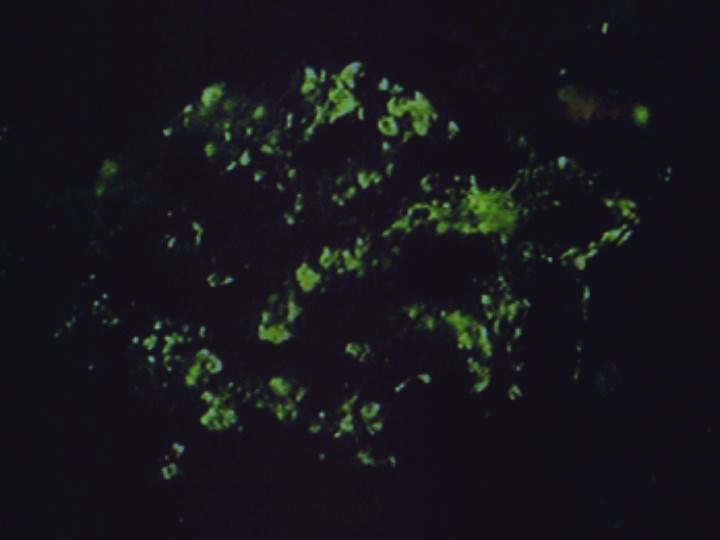
I.F. Granular, diffuse mesangial positivity for C3( 3+ in a scale of 0-3)

Renal biopsy was performed. Light microscopy examination showed mesangial expansion and focal mesangial proliferation on 16 glomerular bodies (Figures 1 and 2), with several mesangial and paramesangial deposits on Acid Fuchsin Orange G (AFOG) stain (Figure 3). No lesions were detected at the tubular level or in the interstitial tissue. In immunofluorescence (IF) study, granular diffuse mesangial positivity for IgA and C3 (3+ in a scale of 0-3) (Figure 4) was observed. A diagnosis of acute renal failure in Lyme disease-associated focal proliferative IgA nephropathy was made. Biopsy was classified as M_1_E_0_S_0_T_1_ (9).

The patient was later discharged, serum creatinine had decreased to 3.5 mg/dl with a BUN of 58 mg/dl, and a slight improvement was observed on follow-up.

## Discussion

The diagnosis of Lyme disease is based primarily on clinical history and on the presence of antibodies (IgM an IgG) to Borrelia burgdorferi. False-positive results have been reported in several infectious diseases such as infectious endocarditis, mononucleosis, CMV infection and other spirochetal diseases such as syphilis and periodontal disease. It has been suggested that the combination of ELISA to detect IgM and IgG anti-B- burgdorferi antibodies and Western blot to confirm the questionable ELISA provides the greatest sensitivity and specificity for the laboratory diagnosis of Lyme disease.

In 2 of the previously reported cases of Lyme-related membranoproliferative glomerulonephritis (MPGN) in humans, an immune-complex related pathogenesis was hypothesised (5,7). In favour of this hypothesis, circulating immune-complexes were detected. In our patient, however, it is possible that Lyme infection could have led to activation of a previous quiescent glomerulopathy. It appears likely that our patient had underlying IgA nephropathy at baseline (chronic ethanolic hepatitis with polyclonal increase of IgA).

The temporal relationship between Lyme infection and acute renal failure raises the probability that the flare was produced by an activation of the immune response.

Our patient’s outcome is fairly satisfactory. He was never placed on renal replacement therapy, as was the case of another patient reported elsewhere (5) and he regained most of his baseline renal function. Renal function improved following treatment for B. burgdorferi infection and owing to the rapid improvement in renal function. Administration of immunoglobulins and corticosteroids was not necessary.

## Conclusion

Borrelia burgdorferi is a possible cause of post-infectious GN in humans as it is in dogs. Difficulties in identifying Borrelia burgdorferi may also be one of the reasons for the paucity of reports on the association of this infection with glomerulonephritis in humans. Currently, various types of histological renal lesions have been reported.

In summary, the clinical course as well as the final outcome of affected patients may vary depending on the severity of renal lesions detected at kidney biopsy.

## Authors’ contributions

LT made diagnosis of Lyme disease-associated focal proliferative IgA nephropathy, CN, AF, PL have contributed to the development of case report and Rolla D revised the manuscript.

## Conflict of interests

The authors have no conflicts of interest to declare.

## Ethical considerations

Ethical issues (including plagiarism, data fabrication, double publication) have been completely observed by the author.

## Funding/Support

This work received no funding from public, commercial, or not-for-profit organizations.
